# Chemokine Receptor 5, a Double-Edged Sword in Metabolic Syndrome and Cardiovascular Disease

**DOI:** 10.3389/fphar.2020.00146

**Published:** 2020-03-03

**Authors:** Zhongwen Zhang, Qiannan Wang, Jinming Yao, Xiaojun Zhou, Junyu Zhao, Xiaoqian Zhang, Jianjun Dong, Lin Liao

**Affiliations:** ^1^ Department of Endocrinology, Shandong Provincial Qianfoshan Hospital, the First Hospital Affiliated with Shandong First Medical University, Jinan, China; ^2^ Division of Endocrinology, Department of Internal Medicine, Shandong Provincial QianFoShan Hospital, Shandong University, Jinan, China; ^3^ Division of Endocrinology, Department of Internal Medicine, Qilu Hospital of Shandong University, Jinan, China

**Keywords:** CCR5, inflammation, endothelial dysfunction, cardiovascular disease, metabolic syndrome

## Abstract

The key characteristic of cardiovascular disease (CVD) is endothelial dysfunction, which is likely the consequence of inflammation. It is well demonstrated that chemokines and their receptors play a crucial role in regulating inflammatory responses, and recently, much attention has been paid to chemokine receptor 5 (CCR5) and its ligands. For example, CCR5 aggravates the inflammatory response in adipose tissue by regulating macrophage recruitment and M1/M2 phenotype switch, thus causing insulin resistance and obesity. Inhibition of CCR5 expression reduces the aggregation of pro-atherogenic cytokines to the site of arterial injury. However, targeting CCR5 is not always effective, and emerging evidence has shown that CCR5 facilitates progenitor cell recruitment and promotes vascular endothelial cell repair. In this paper, we provide recent insights into the role of CCR5 and its ligands in metabolic syndrome as related to cardiovascular disease and the opportunities and roadblocks in targeting CCR5 and its ligands.

## Introduction

Metabolic syndrome (MetS), including obesity, hypertension, hyperglycemia, and dyslipidemia, has detrimental effects on the endothelium, contributing to the development of cardiovascular diseases (CVD) (Nikolopoulou and Kadoglou, 2014; Yao et al., 2014). One of the key common central mechanisms linking all of these diseases is underpinned by an exaggerated inflammatory response (Lumeng et al., 2007). In recent years, evidence has accumulated that chemokine receptor 5 (CCR5) and its ligands play a critical role in regulating the inflammatory response. For example, CCR5 aggravates the inflammatory response in mouse adipose tissue by regulating macrophage recruitment and M1/M2 phenotype switching, thus causing insulin resistance and obesity (Kitade et al., 2012). Inhibition of CCR5 expression reduces the accumulation of pro-atherogenic cytokines and monocytes to the site of arterial injury. However, therapies targeting CCR5 and its ligands have not performed consistently with regard to preventing metabolic syndrome related diseases (Kennedy et al., 2013; Slominski et al., 2017), indicating that CCR5 and its ligands might play a double-edged role in the progression of these diseases. More importantly, emerging evidence shows that CCR5 is specifically expressed in endothelial cells and endothelial progenitor cells (EPCs). CCR5 facilitates progenitor cell recruitment and promotes vascular endothelial repair in a mouse model (Ishida et al., 2012; Suffee et al., 2012; Zhang et al., 2015b). Although inhibiting CCR5 expression reduces the inflammatory response, it also aggravates the endothelial damage, thus significantly limiting the actual effectiveness of therapeutic interventions. Therefore, studying the mechanisms of CCR5 and its ligands that control these processes in the endothelial cells and the inflammatory response will provide further understanding of the pathophysiology of cardiovascular disease and may be used to develop novel pharmacological strategies.

## CCR5 and Its Ligands

CCR5, a member of the guanine nucleotide binding protein (G protein) coupled receptors (GPCR), has been known as a key player in HIV-1 entry into target cells from its discovery (Berger et al., 1999). CCR5 binds and responds to chemokine ligand 3 (CCL3) (**Table 1**), chemokine ligand 4 (CCL4), and chemokine ligand 5(CCL5). CCR5 is expressed in macrophages, activated T cells, natural killer cells, endothelial cells, and EPCs. CCR5 participates in the regulation of proinflammatory response by modulating the behavior, survival, and retention of immune cells in tissues (Kohlmeier et al., 2011). In addition, CCR5 can be expressed in non-immune system cells, notably in astrocytes, microglia, and neurons, which are involved in neuronal survival and differentiation (Sorce et al., 2011).

**Table 1:** Summary of data of CCR5 and its ligands, primary source, main effects, and main references.

Gene name	Expressed by/primary source	Main effects		References
		Pro-inflammation	Endothelium repair and angiogenesis	
**CCL3**	Monocytes/macrophages, T cells, vascular smooth muscle cells, eosinophils, coronary endothelial cells, and platelets.	Mediates the recruitment of macrophages into the injured site by binding with its receptor, CCR5.	CCL3 induces the infiltration of macrophages into the damaged retina and produces vascular endothelial growth factor (VEGF) by binding to CCR5, and eventually promotes corneal neovascularization.	Ridiandries et al., 2016; Menten et al., 2002; DiPietro et al., 1998; Lu et al., 2008.
**CCL4**	Monocyte, T cells, B lymphocytes, NK cells, dendritic cells, vascular smooth muscle cells, and neutrophils.	Chemoattractants for immature dendritic cells and macrophages/monocytes, attracts macrophages to destroy islet cells.	Increases VEGF-C expression and promotes lymph angiogenesis in oral cancer cells.	Ridiandries et al., 2016; Menten et al., 2002; Chang and Chen, 2016; Lien et al., 2018.
**CCL5**	T-cells, epithelial cells and activated platelets	Mediates the macrophage recruitment and M1/M2 phenotype switching, recruits leukocytes and certain natural-killer cells, promotes smooth muscle cells phenotypic switching from the contractile to synthetic phenotype.	CCL5 is pro-angiogenic in the ischemic tissues and subcutaneous model, promotes the revascularization and muscle regeneration by binding to its receptor, CCR5.	Suffee et al., 2012; Liu et al., 2014; Zhang et al., 2015b; Ridiandries et al., 2016; Lin et al., 2018.
**CCR5**	Monocytes/macrophages, activated T cells, endothelial cells, endothelial progenitor cells (EPCs), natural killer cells, astrocytes, microglia, and neurons.	Promotes infiltration of monocytes/macrophages to the injured site, aggravates hepatic steatosis and insulin resistance, and increases triglyceride synthesis.	Accelerates the homing of EPCs to damaged endothelial cells, promoting endothelial repair or the formation of neovascularization.	Suffee et al., 2017; Bjerregaard et al., 2019; Rookmaaker et al., 2007; Potteaux et al., 2006; Berres et al., 2010; Ishida et al., 2012; Kitade et al., 2012; Shen et al., 2013; Liu et al., 2014; Zhang et al., 2015b; Ridiandries et al., 2016; Perez-Martinez et al., 2018; Yan et al., 2019.

CCL3, also known as macrophage inflammatory protein-α (MIP-α), is released from activated platelets, mast cells, and neutrophils (Weber, 2005; Montecucco et al., 2008). Previous studies indicated that CCL3 activates neutrophils *via* the mediation of firm adherence and the (subsequent) transmigration of neutrophils as a result of lipid mediator production. CCL4 is also called macrophage inflammatory protein-β (MIP-β) and was first isolated from culture medium containing lipopolysaccharide-activated macrophages. CCL4 can induce the chemotaxis of different cell types, including natural killer cells, monocytes/macrophages, and coronary endothelial cells (Mirabelli-Badenier et al., 2011). The chemotactic activity of CCL5, initially considered to be a T cell-specific protein that is stored in and released from various cells, including endothelial cells, EPCs, monocytes/macrophages and fibroblasts, recruits activated T cells, NK cells, and basophils to the site of an inflammatory response (Appay and Rowland-Jones, 2001).

## CCR5 and Its Ligands in Relation to Endothelial Function

Endothelial cells line the interior surface of all blood vessels and are involved not only in delivering blood to all vital organs but also in maintaining the homeostasis of the vasculature. A large body of evidence has shown that diabetes, ischemia, and atherosclerosis (Suffee et al., 2012; Zhang et al., 2015b; Ali and Woodman, 2019) have adverse effects on the endothelium, which contributes to the development of CVD. One of the key common central mechanisms linking all of these diseases is based on exaggerated inflammation. In all cases, the interaction between the endothelium and inflammatory cells plays a key role in the initiation of the pathological condition.

Previous studies have demonstrated that chemokines can directly regulate the migration and recruitment of cells to injury sites *via* inflammation. All CC-chemokines contain nuclear factor-kappa B (NF-κB) binding motifs, and their expression is significantly upregulated under inflammatory conditions (Werts et al., 2007; Ridiandries et al., 2016). CCL3, CCL4, and CCL5 are upregulated when induced by an inflammatory stimulus (Laurence, 2006; Zhang et al., 2015a; Ridiandries et al., 2016). Increased expression of CCL3/CCL4/CCL5 mediates the arrest and transmigration of monocytes/macrophages into the damaged endothelium by binding with its receptor CCR5 (Zhang et al., 2015b; Ridiandries et al., 2016), which is involved in the inflammatory response to endothelial injury. Blocking CCR5 alleviated myocardial ischemia–reperfusion injury in rats by regulating the cardiac inflammatory response (Shen et al., 2013). CCR5 deficiency could reduce macrophage aggregation into atherosclerotic plaques in a hypercholesterolemic mouse model (Potteaux et al., 2006).

In addition to their roles in mediating inflammation, CCL5 has also been shown to play a role in the process of ischemia-mediated physiological angiogenesis (Suffee et al., 2017; Bjerregaard et al., 2019) and endothelial repair (Maarten B. et al., 2007; Zhang et al., 2015b; Yan et al., 2019). CCL5/CCR5 is specifically expressed in endothelial cells and EPCs, and endothelial cell specific CCR5 is involved in the regulation of vascular regeneration in ischemic tissues (Suffee et al., 2017). Administration of CCL5-loaded microparticles could improve the clinical score of mice after limb injury as well as promote the revascularization and the muscle regeneration. Yan et al. (2019) verified that CCR5 expression was upregulated in vascular endothelial growth factor (VEGF) modified macrophage *in vitro* after treatment with VEGF-modified macrophages therapy accelerated reendothelialization and attenuated neointima formation in the wire-induced carotid artery injury mouse model. CCL5 is pro-angiogenic in a rat model of subcutaneous injury. One *in vitro* study found that the effects of CCL5-mediated angiogenesis are at least partially dependent on VEGF secretion by endothelial cells, as the effects are weaker when endothelial cells are incubated with anti-VEGF receptor antibodies (Suffee et al., 2012). According to the leucocyte subset chemokine expression, patients with age-related macular degeneration (AMD) of neovascularization have different responses to anti-VEGF receptor antibody treatment, with good responders to the anti-VEGF loading dose having higher CCR1 expression on monocytes and lower CCR5 expression on CD14^+^ T cells, indicating that CCR5 may be an effective way to provide individualized treatment for neovascular AMD (Bjerregaard et al., 2019).

EPCs, as a kind of precursor cell derived from bone marrow that can differentiate into endothelial cells, play an important role in neovascularization during tissue repair (Zhang et al., 2015b). In a mouse skin injury model, deletion of the CCR5 gene reduced the accumulation of vascular EPCs and the formation of neovascularization, and it eventually delayed the healing of damaged skin. When EPCs carrying the CCR5 gene are transferred into CCR5^-/-^ mice, EPCs accumulated at the site of injury and restored normal neovascularization (Ishida et al., 2012). CCL5 is involved in the homing of bone marrow-derived EPCs in glomerular endothelial repair. In a mouse model of reversible glomerulonephritis, administration of a CCR5 inhibitor (METRANTES) reduced the participation of EPCs in glomerular vascular repair (Rookmaaker et al., 2007). In a hypercholesterolemic ApoE^-/-^ mouse model, overexpression of CCR5 contributes to the homing of EPCs to damaged endothelial cells, promoting endothelial repair, improving endothelial dysfunction, and ultimately stabilizing atherosclerotic plaques (Zhang et al., 2015b).

CCR5 and its ligands play an important role in regulating tissue angiogenesis, but the exact mechanism is still unclear. A study (Liu et al., 2014) on human chondrosarcoma cells revealed that pretreatment with a phosphatidylinositol 3-kinase (PI3K) inhibitor repressed the VEGF production and angiogenesis induced by CCL5/CCR5, suggesting that the PI3K-dependent pathway plays a crucial role in CCL5/CCR5-mediated angiogenesis.

In addition to CCL5, CCL3 and CCL4 are also involved in the process of revascularization. In alkali-induced corneal neovascularization of mouse models, CCL3 could induce the infiltration of macrophages into the damaged retina and the production VEGF by binding to CCR5, eventually promoting corneal neovascularization (Lu et al., 2008). Anti-CCL3 antiserum has been shown to decrease angiogenic activity in a murine wound repair model (DiPietro et al., 1998). CCL4 was proven to increase VEGF-C expression and promote lymphangiogenesis in oral cancer cells (Lien et al., 2018).

## CCR5 and Its Ligands in Metabolic Syndrome

### Obesity

Obesity is characterized as low-grade systemic or chronic inflammation that is associated with an increased incidence of metabolic syndrome, cardiovascular disease, and tumor (Despres and Lemieux, 2006; Nikolopoulou and Kadoglou, 2014). Excessive fat tissue expansion triggers the secretion of cytokines and chemokines (Brownlee, 2005), which in turn attract various leukocytes, leading to fatty tissue inflammation.

The exact role of CCR5 and its ligands in the pathogenesis of obesity is still obscure, but there are several studies that have continuously reported this finding (Yao et al., 2014). Gao et al. (2015) noted that phosphatidyl-ethanol-amine-N-methyl transferase-deficient mice were resistant to high fat diet-induced obesity. This may result from decreased expression of CCL5. Similarly, Pisano et al. (2017) found that weight gain in patients on antipsychotics is associated with the extent of CCL5 expression. CCL5, as a neuroendocrine element, modulates food intake and body temperature of C57BL/6 mice through unidentified receptors in the hypothalamus (Chou et al., 2016), thus affecting the body weight. In a high-fat diet-induced obese mouse model (Kitade et al., 2012), CCR5 plays a critical role in adipose tissue macrophage recruitment and polarization. Deletion of CCR5 reduces the transition of macrophages from the pro-inflammatory M1 phenotype to the anti-inflammatory M2 phenotype and ameliorated obesity-induced insulin resistance. This observation is consistent with previous studies that have indicated that CCR5 could directly induce the transition of the M1/M2 phenotype by modulating the alteration of Ly6C ^high^ and Ly6C ^low^ monocyte subsets (Soehnlein et al., 2013; Huh et al., 2018); further studies are required to clarify the details of this mechanism.

### Hyperglycemia

CCR5 and its ligands have been shown to be associated with the pathogenesis of both type 1 diabetes mellitus (T1DM) and type 2 diabetes mellitus (T2DM). As we know, the main pathological mechanism of T1DM is the pancreatic islet β-cell death (Ashcroft and Rorsman, 2012). CCL4 is upregulated in the islet autoantibody-positive T1DM patients and relatives at high risk of developing T1DM, and increased expression of CCL4 aggravates β-cell death and early islet graft loss by stimulating the trafficking of macrophages into injured pancreatic islets (Hanifi-Moghaddam et al., 2006). Moreover, the extracellular regulated protein kinases and NF-kB pathway may be involved in the process of CCL4 production by stimulating CD40-CD40L interaction in human pancreatic islets (Barbe-Tuana et al., 2006).

In patients with diabetes, CCL5 and CCR5 are upregulated in the peripheral blood (Slominski et al., 2019; Inayat et al., 2019). Exogenous insulin supplementation may reduce concentrations of CCL5 in patients with newly diagnosed type 2 diabetes compared with the control subjects (Bogdanski et al., 2007). Epidemiological studies have indicated that CCR5 promoter function mutation (CCR5 59029 G to A alteration) could be a susceptibility factor for type 2 diabetes and the CCR5 59029 A positive genotype increased the risk for type 2 diabetes (Kochetova et al., 2019). The exact mechanism of CCR5 gene mutation and the pathogenesis of type 2 diabetes are still unclear. Since the metabolic hallmark of type 2 diabetes is insulin resistance, previous studies have shown that CCR5 gene knockout in mice could prevent insulin resistance and diabetes induced by a high-fat feeding. The beneficial effects of CCR5 deficiency were correlated with reduced recruitment and the M2-dominant shift of macrophages in adipose tissues (Kitade et al., 2012). However, this finding is contrary to Chou et al’ s studies, which suggested that the CCR5 gene knockout in mice impairs the regulation of energy metabolism in the hypothalamus (Chou et al., 2016). Both *in vitro* tissue culture and *ex vivo* stimulation studies indicated that the activation of PI3K-Akt pathways and insulin signaling were impaired in the hypothalamus of CCR5 knockout mice. METRANTES, a CCR5 antagonist, abolished the dephosphorylation of insulin receptor substrates-1 (IRS-1)^S302^ and insulin signal activation. In addition, intracerebroventricular delivery of the CCR5 antagonist interrupted hypothalamic insulin signaling and led to glucose intolerance. In summary, CCR5 may be involved in the pathogenesis of type 2 diabetes through mediating insulin resistance and hypothalamic insulin signaling regulation. However, there are still many unanswered questions about the exact effect of CCR5 in the pathogenesis of type 2 diabetes. Additional research is needed in the future to confirm this conclusion.

Microvascular complications are the leading cause of death in diabetic patients. The recruitment of leukocytes to kidney tissue during T2DM is an early event in the pathogenesis of diabetic kidney disease (DKD). CCR5 mRNA was faintly detected in the normal tubulointerstitial compartment tissue (Mezzano et al., 2003). After the high glucose treatment, CCR5 expression was upregulated in the tubulointerstitial compartment during the process of diabetes. Since CCL5/CCR5 participates in the formation of inflammatory infiltrates during glomerulonephritis, inhibition of CCR5 exerts renal protection during early glomerulonephritis through its anti-inflammatory properties (Turner et al., 2008). The correlation between the CCR5 gene and the risk of DKD is conflicting and inconclusive. An oral CCR2/CCR5 antagonist (PF-04634817) slightly reduced albuminuria in adults with DKD (Gale et al., 2018). However, in ob/ob mice, treatment with a dual CCR2/CCR5 antagonist (MK-0812) showed no protective effect on DKD (O'Brien et al., 2017). Although adipose tissue inflammation was decreased in this mouse model, the improvement was insufficient to overcome the metabolic imbalances of type 2 diabetes. The mutations in the CCR5 gene promoter region (CCR5 59029 G to A alteration) and deletion of 32 nucleotides (CCR5-Δ32) lead to genetic inactivation of CCR5 (Nazir et al., 2014). Previous studies have found that CCR5-59029 G/A was an independent risk factor for DKD (Yahya et al., 2019). The CCR5 59029A-positive genotype was correlated with an increased risk for albuminuria (Zhang et al., 2016). Mlynarski et al. (2005) showed that the CCR5-Δ32 mutation increased the risk of kidney disease in men with type 1 diabetes; however, this outcome is contrary to that of Prasad et al. (2007) who found that CCR5-Δ32 was not related to nephropathic type 2 diabetes patients. Skrzypkowska et al. (2019) even indicated that 32 allele-bearing individuals exhibit more beneficial values of kidney function parameters. Specifically, the wt/∆32 and ∆32/∆32 carriers exhibited a higher number of CD34^+^VEGFR^2+^ and CD34^+^VEGFR^2+^c-Kit^+^ cells than that in the wild type counterparts.

In addition, CCR5-Δ32 gene mutation was associated with retinopathy in patients with type 1 diabetes. Previous studies indicated that tumor necrosis factor (TNF)-α, vascular cell adhesion molecule (VCAM)-1, and intercellular cell adhesion molecule (ICAM)-1 were upregulated in diabetic patients with *CCR5*-Δ32 carriers (Joussen et al., 2002; Slominski et al., 2017). TNF-α plays a major role in the degeneration of retinal capillaries. Since ICAM-1 is the primary adhesion molecule involved in the pathogenesis of diabetic retinopathy (DR), the elevated level of ICAM-1 may facilitate the recruitment of leukocytes into the damaged retina (McLeod et al., 1995). Thus, the mutation of CCR5 gene (CCR5-Δ32) in the retina may lead to upregulating expression of other cytokines that exacerbate retinal damage.

### Dyslipidemia

Diabetes is often accompanied by dyslipidemia as a result of insulin resistance. Dyslipidemia has been demonstrated to be detrimental to diabetes microvascular and macrovascular complications (Cooper and Jandeleit-Dahm, 2005; Margeirsdottir et al., 2008).

Clinical research has shown that the expression levels of CCL5 and CCR5 were increased in the subcutaneous adipose tissue of obese individuals in comparison with those in the lean population, which could be reduced back to the normal levels through physical exercise (Baturcam et al., 2014). One study (Kim et al., 2018) reported that ultraviolet irradiation of human sun-protected subcutaneous fat *in vitro* could induce CXCL5 and CCL5 production; CCL5 treatment dose-dependently reduced triglyceride (TG) content and downregulated the expressions of acetyl CoA carboxylase (ACC), fatty acid synthase (FAS), stearoyl CoA desaturase (SCD), and sterol regulatory element-binding protein-1(SREBP-1) in human adipocytes. The changes could be reversed when the CCL5 receptor, the CCR5 gene, is deleted, suggesting that CCL5 impairs the synthesis of TG by reducing the expression of SREBP-1 and lipogenic enzymes through binding to its receptor, CCR5. In a nonalcoholic fatty liver disease mouse model, treatment with a CCR5 antagonist, maraviroc, could ameliorate hepatic steatosis *via* downregulation of dietary lipid absorption or *de novo* lipogenesis (Perez-Martinez et al., 2018). This also accords with Kitade et al.’s (2012) earlier observations, which showed that the deletion of the CCR5 gene reduced the content of TG and the lipogenic genes expression in mice. Similarly, Berres et al. (2010) even found that the administration of CCR5 antagonist markedly ameliorated hepatic fibrosis and accelerated fibrosis regression in mouse models of liver fibrosis. Epidemiological research (Hyde et al., 2010) has also shown that there was a significant positive correlation between the CCR5-Δ32 mutation and elevated serum high-density lipoprotein cholesterol (HDL) and reduced serum TG, both of which are beneficial from a cardiovascular perspective.

In addition to endothelial cells, dyslipidemia could drive the phenotypic modulation of smooth muscle cells (SMCs) and cause SMCs phenotypic alteration from the physiologically contractile to the pathophysiologically synthetic phenotype. CCR5 and CCL5 play crucial roles in the phenotypic modulation of SMCs. In HFD fed mouse model, the CCR5 and CCL5 gene knockouts showed significantly decreased levels of serum lipids and increased expressions of the SMCs contractile phenotype in the thoracoabdominal aorta as compared with the levels observed in wild-type mice. *In vitro*, CCL5 treated human aorta derived SMCs could induce cell proliferation and promote the phenotypic switching from the contractile to the synthetic phenotype (Lin et al., 2018).

### Hypertension

Hypertension is an important risk factor for the development of cardiovascular diseases (Perticone et al., 2004). Previous studies have shown that angiotensin II (Ang II) promotes the infiltration of T cells and monocytes into perivascular adipose tissues (pVAT) (Mikolajczyk et al., 2016). Subsequent studies have demonstrated that the activation and recruitment of T cells and monocytes into pVAT is very important in the pathogenesis of renin-angiotensin system (RAS)-dependent hypertension (Guzik et al., 2007).

CCL5 is produced by several tissues that contribute to the regulation of the vasoconstriction and diastolic function, such as the vascular endothelium, vascular smooth muscle (Jordan et al., 1997), glomeruli (Wolf et al., 1997), renal tubules (Wada et al., 1999), and the central nervous system (Gouraud et al., 2011). CCL5 expression is upregulated in the aorta and pVAT during RAS-dependent hypertension. Previous studies have shown that there is a significant positive correlation between CCL5 expression and blood pressure in the Ang II-induced hypertension mouse model (Mikolajczyk et al., 2016). CCL5 could enhance the genesis of perivascular inflammation, thus affecting the development of hypertensive vascular dysfunction. The deletion of CCL5 reduces the infiltration of leukocytes and T lymphocytes into pVAT and importantly, this is independent of blood pressure changes.

Furthermore, the effects of CCL5 signaling on hypertensive organ damage appear to be tissue-and context-dependent. For example, CCL5 and CCR5 are possibly involved in the pathogenesis of pulmonary arterial hypertension (PAH). CCL5 can be released from endothelial cells and perivascular fibroblasts. Anti-endothelial cell antibody (AECA)-positive systemic sclerosis patients are associated with an increased risk of PAH, which may result from the increased CCL5 expression induced in endothelial cells by the stimulation with AECA. CCR5 is expressed in the macrophages, pulmonary artery endothelial cells, and pulmonary artery smooth muscle cells. Inhibition of CCR5 expression in mice model decreased perivascular macrophages recruitment and the proliferation of pulmonary-artery smooth muscle cell during hypoxia exposure (Mamazhakypov et al., 2019). However, this finding is contrary to previous studies which have suggested that CCL5 plays an important protective role in hypertension-induced renal injury. In the angiotensin II-induced hypertension mice model, CCL5 gene deficiency exhibited markedly aggravation of kidney damage, macrophage infiltration, and proinflammatory cytokine expression, which led to the aggravation of urinary albumin excretion (Rudemiller and Crowley, 2017). This may be explained by the blockade of one chemokine leading to the upregulated expressions of other cytokines that exacerbate RAS-dependent hypertension, as CCL2 blockade abrogates the enhanced renal macrophage infiltration and interstitial fibrosis in CCL5-deficient mice (Mikolajczyk et al., 2016; Rudemiller et al., 2016).

### Atherosclerosis

Atherosclerosis is characterized by the accumulation of lipids, immune cells, and cell debris in the vessel wall, which form atherosclerotic lesions that can grow over time and eventually occlude the blood vessels, leading to ischemia and angina (Halvorsen et al., 2015; Pothineni et al., 2017). As a chronic inflammatory disease, atherosclerosis is associated with many chemokines and chemokine receptors (Zhang et al., 2015b; Andersen et al., 2019; Van der Vorst et al., 2019).

In recent years, much attention has been focused on the role of CCR5 and its ligands, which is crucial in the context of atherosclerosis initiation and progression (Pai et al., 2006; Afzal et al., 2008; Zhang et al., 2015a). CCR5 is expressed in the endothelial cells, monocytes/macrophages, and leukocytes (Soehnlein et al., 2013). CCL5 may be released from activated platelets and T cells. During the progression of atherosclerotic disease, CCR5 and CCL5 proteins are faintly detected when there are no visible atherosclerotic plaques and are highly expressed in the stable plaques and advanced unstable plaques (Zhang et al., 2015b). Activated platelets release CCL5 (Gleissner et al., 2008), transferring CCL5 to the surface of injured endothelial cells and leading to increased monocyte/macrophage and leukocyte adhesion to the atherosclerotic vascular wall by binding with its receptor, CCR5 (Soehnlein et al., 2013; Zhang et al., 2015b), both of which are adverse effects from a cardiovascular perspective. The deletion of the CCR5 gene in apolipoprotein E-deficient (ApoE^-/-^) mice has a protective effect on diet-induced atherosclerosis and reduces the infiltration of mononuclear and Th1 type immune response. CCR5 is also associated with a more stable plaque phenotype (Braunersreuther et al., 2007). Administration of the CC chemokine antagonist METRANTES (Veillard et al., 2004) or treatment with [^44^AANA^47^]-RANTES (Braunersreuther et al., 2008) inhibits the progression of atherosclerosis in a hyperlipidemic mouse model. This inhibition of lesions is associated with reduced infiltration of leukocytes into plaques and increased content of smooth muscle cells and collagen content, indicating a more stable plaque phenotype. Systemic CCL5 deficiency in ApoE^-/-^ mice was found to cause reduced neointima formation after carotid artery injury (Czepluch et al., 2016). The atheroprotective effect of CCL5 deficiency might be mediated by the upregulation of kruppel-like factor 4 expression in smooth muscle cells. For HIV-infected patients, treatment with a CCR5 antagonist (Maraviroc) could significantly improve endothelial dysfunction, arterial stiffness, and early carotid atherosclerosis (Francisci et al., 2019). Matrix metalloproteinases (MMP) are involved in the vascular remodeling and immunomodulation during the process of atherosclerosis (Clemente et al., 2018). Studies have demonstrated that mice deficient for MT4-MMP have higher numbers of patrolling monocytes/macrophages adhered to inflamed endothelial cells, leading to larger lipid deposits in atherosclerotic plaques. Interestingly, these effects could be reversed by CCR5 inhibition (Clemente et al., 2018). However, epidemiological studies have shown that the association between CCR5 gene mutation and the risk of atherosclerosis-related diseases is conflicting and inconclusive. Previous studies have found that CCR5-Δ32 allele bearing individuals exhibit more beneficial values of cardiovascular function parameters (Pai et al., 2006; Afzal et al., 2008; Hyde et al., 2010). There was a significant positive correlation between CCR5-Δ32 allele bearing individuals and reduced susceptibility to CVD (Afzal et al., 2008) or development of CVD in a North Indian population (Pai et al., 2006). This may be due to CCR5 deficiency affecting lipid metabolism (Hyde et al., 2010). CCR5-Δ32 was significantly associated with higher levels of HDL-C and lower levels of TG, both of which are beneficial from a cardiovascular perspective. However, this outcome is contrary to those of Sharda et al. (2008) and Apostolakis et al. (2007) who found that there was no significant difference between the CCR5-Δ32 and the risk of CVD. Zhang et al. (2015a) even indicated that the CCR5-Δ32 increased the risk of atherosclerotic disease in Asian population (Zhang et al., 2015a). Moreover, previous studies have reported that a CCR5 gene promoter region mutation (CCR5-59029 G/A) was an independent risk factor for CVD (Simeoni et al., 2004; Vogiatzi et al., 2009). The CCR5 59029A-positive genotype was correlated with an increased risk of acute coronary syndrome (Ting et al., 2015). Considering that the role of CCR5 gene mutation in the risk of CVD is controversial, further studies with more focus on the association is therefore suggested.

In addition to CCL5, CCL3 and CCL4 have also been reported to participate in the process of atherosclerosis. CCL3 was highly expressed in atherosclerotic plaques, and the treatment with atorvastatin alleviated atherosclerotic lesions through inhibition of the 5-Lipoxygenase pathway and downregulation of CCL3 expressions in an atherosclerotic mouse model (Yang et al., 2013). CCL4 was also upregulated in vulnerable atherosclerosis plaques and was expressed by T cells in advanced atherosclerotic lesions in stroke patients (Montecucco et al., 2010). Studies has been conducted in a cohort of hypertensive patients for an average follow-up period of 37.2 ± 19.9 months; the result found that elevated serum CCL4 levels is an independent predictor of stroke and cardiovascular events (Tatara et al., 2009).

## Discussion

**Figure 1:**
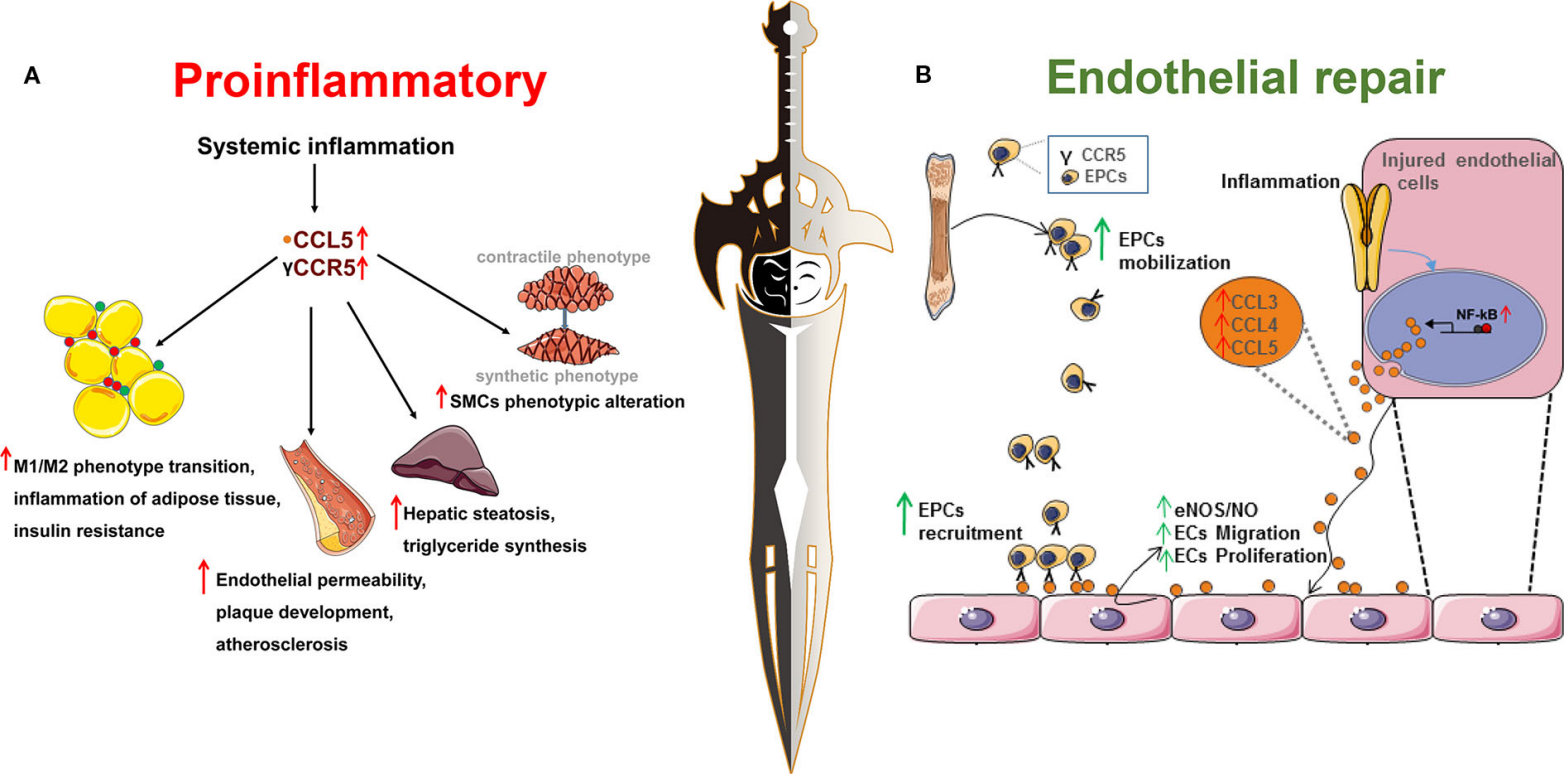
Chemokine receptor 5, a double-edged sword in the inflammatory response and endothelial repair during the process of metabolic syndrome and cardiovascular disease. **(A)** The proinflammatory role of CCR5 and its ligands in metabolic syndrome and cardiovascular disease. As shown in section **(A)**, obesity is characterized as low-grade systemic or chronic inflammation that is associated with increased incidence of metabolic syndrome and cardiovascular disease. CCR5 and its ligands are associated with systemic inflammation. (1) CCR5 and its ligands promote the transition of macrophages from the pro-inflammatory M1 phenotype to the anti-inflammatory M2 phenotype and aggravate obesity-induced insulin resistance; (2) CCR5 and its ligands promote infiltration of leukocytes into plaques and endothelial permeability, decrease the content of smooth muscle cells and collagen content, indicating a more vulnerable plaque phenotype; (3) CCL5 increases the synthesis of triglyceride and hepatic steatosis through binding to its receptor, CCR5; (4) CCL5 could induce smooth muscle cell proliferation and promote the phenotypic switching from the contractile to the synthetic phenotype; **(B)** CCR5 and its ligands are involved in the endothelial repair during the process of endothelial damage. As shown in section **(B)**, CCL3, CCL4, and CCL5 contain NF-κB binding motifs and are upregulated when induced by an inflammatory stimulus. Increased expression of CCL3/CCL4/CCL5 mediates the mobilization and recruitment of bone marrow derived-endothelial progenitor cells into the damaged endothelium by binding with its receptor, CCR5. In addition, CCL3, CCL4, and CCL5 could directly stimulate injured cells, increase nitric oxide production, and promote endothelial cell migration and proliferation to the injured sites.

It is generally assumed that CCR5 and its ligands play a critical role in promoting inflammation by recruiting immune cells, such as monocytes and T cells. They may contribute to insulin resistance by M1/M2 phenotype switching of macrophages that infiltrate adipose tissues (Kitade et al., 2012). On the one hand, an impaired insulin signal *via* PI3K-Akt directly reduces endothelial NO synthase (eNOS) activation (Rask-Madsen and King, 2007), leading to endothelial dysfunction. On the other hand, long-term exposure of endothelial cells to high levels of glucose induces cellular dysfunction (Brownlee, 2005) and production of CCR5 and its ligands (Li et al., 2013). Adipokines, primarily adiponectin and TNF-α, secreted by fat tissue, also contribute to endothelial damage (Rask-Madsen and King, 2007), which is regarded as the initiation of cardiovascular diseases. CCR5 seems to be associated with endothelial dysfunction *via* proinflammatory activity (**Figure 1A**) (Yao et al., 2014).

However, therapies targeting CCR5 and its ligands are not always satisfactory. Populations with CCR5-Δ32 are not consistently protected from diabetes and its complications. Deletion of CCR5 or treatment with a CCR5 antagonist in mouse model did not always reverse the inflammatory status in metabolic syndrome. One possible explanation may be that blockade of one chemokine leads to the upregulated expressions of other cytokines that exacerbate cellular dysfunction and inflammation. Another explanation may point to the potential therapeutic effect role of CCR5 in endothelial repair. The macrophages recruited by CCR5 may release various pro-angiogenic factors, including VEGF, basic fibroblast growth factor (bFGF), and platelet derived growth factor (PDGF) (**Figure 1B**) (Ridiandries et al., 2016). In addition, CCR5 facilitates the recruitment of EPCs into injured vessels and enhances endothelial regeneration, which may also explain the genetic inactivation of CCR5 as an independent risk factor for DR and DKD (Slominski et al., 2017).

CCR5 is a double-edged sword for metabolism-related cardiovascular diseases, which may result from the patients with varying degrees of damage at different growth stages. In addition, the specificity of populations and organs should also be taken into consideration. Further studies are required to clarify the details of the mechanism.

## Author Contributions

LL and JD contributed to study conception and design, literature review, and preparation of the manuscript. JY, XZho, JZ, and XZha contributed to study conception and design. ZZ and QW drafted the manuscript, revised it critically for important intellectual content, gave final approval of the version to be sent, and read and approved the final manuscript.

## Funding

This work was supported by the Key Research & Development Plan of Shandong Province (No. 2018GSF118176), the Natural Science Foundation of Shandong Province (No. ZR2016HQ26), the National Natural Science Foundation of China (81670757, 81770822), and the Bethune-Merck's Diabetes Research Foundation (No. G2016014).

## Conflict of Interest

The authors declare that the research was conducted in the absence of any commercial or financial relationships that could be construed as a potential conflict of interest.
